# Plant growth-promoting traits of *Pseudomonas geniculata* isolated from chickpea nodules

**DOI:** 10.1007/s13205-014-0263-4

**Published:** 2014-11-01

**Authors:** Subramaniam Gopalakrishnan, Vadlamudi Srinivas, Bandikinda Prakash, Arumugam Sathya, Rajendran Vijayabharathi

**Affiliations:** International Crops Research Institute for the Semi-Arid Tropics (ICRISAT), Patancheru, 502 324 Telangana India

**Keywords:** Plant growth promotion, *Pseudomonas geniculata*, Chickpea, Field evaluation

## Abstract

A bacterium, isolated from nodules of chickpea grown in alluvial soils of Haryana state of India, designated as IC-76 was characterized for in vitro plant growth-promoting (PGP) properties and further evaluated under greenhouse, on-station and on-farm field conditions for PGP activity in chickpea. The isolate IC-76 produced indole acetic acid, siderophore, hydrocyanic acid, cellulase, protease, and β-1,3-glucanase. When the bacterium was evaluated individually for their PGP potential in the greenhouse on chickpea and in combination with five *Streptomyces* sp. (strains CAI-24, CAI-121, CAI-127, KAI-32, and KAI-90; demonstrated earlier as biocontrol potential against *Fusarium* wilt disease in chickpea), the traits, including nodule number and weight, shoot, and root weight, pod number and weight, seed number and weight, available phosphorus and  % organic carbon were found significantly, enhanced over un-inoculated control. In the on-station and on-farm field conditions, IC-76 significantly enhanced nodule number and weight, shoot, and root weight, stover and grain yield and total dry matter. In the rhizosphere (0–15 cm soil), the bacterium also significantly enhanced the total nitrogen, available phosphorus and  % organic carbon. The sequence of 16S rDNA gene of the IC-76 was matched with *Pseudomonas geniculata* in BLAST analysis. This study demonstrates that IC-76 has the potential for PGP in chickpea.

## Introduction

Plant growth-promoting (PGP) microbes are soil bacteria that colonize rhizoplane and rhizosphere and enhance plant growth when inoculated artificially onto the soil or seeds. PGP bacteria can directly or indirectly affect plant growth through various mechanisms which includes fixation of atmospheric nitrogen (Soares et al. 2006), solubilization of minerals (Basak and Biswas 2009; Panhwar et al. 2012), synthesis of various enzymes and phyto-hormones (Patten and Glick 2002; Cheng et al. 2007), and inhibition of phytopathogens (Hao et al. 2011; Gopalakrishnan et al. 2011a, b, c). Different functional and taxonomic groups of microbes are reported to have PGP traits which includes free living bacteria, such as *Bacillus*, *Pseudomonas*, *Erwinia*, *Caulobacter*, *Serratia*, *Arthrobacter*, *Micrococcus*, *Flavobacterium*, *Chromobacterium*, *Agrobacterium*, *Hyphomicrobium*, and symbiotic N_2_ fixing bacteria, such as *Rhizobium*, *Bradyrhizobium*, *Sinorhizobium*, *Azorhizobium*, *Mesorhizobium*, and *Allorhizobium* (Vessey 2003). The use of PGP microbes has increased in many parts of the world due to their significant contribution in growth and yield which has been demonstrated in crops, such as tomato, wheat, rice, bean, and pea (Tokala et al. 2002; Nassar et al. 2003; El-Tarabily 2008; Figueiredo et al. 2008; Sadeghi et al. 2012; Gopalakrishnan et al. 2012a, b, 2013a, b). In legumes, symbiotic bacteria including *Rhizobium*, *Mesorhizobium*, and *Bradyrhizobium* are reported to benefit plant growth and yield (Pandey and Maheshwari 2007; Joshi et al. 2008; Kumar et al. 2011). Besides, several non-symbiotic bacteria, including *P*s*eudomonas*, *Bacillus*, *Klebsiella*, *Azotobacter*, *Azospirillum*, and *Azomonas* were also reported to increase the plant growth by the same mechanisms followed by *Rhizobium* sp. (Glick 1995; Ahemad and Kibret 2014). Number of other bacteria such as *Pantoeaagglomerans*, *Klebsiellapneumoniae*, *Beijerinckia* sp., and *Azoarcus* sp. were also reported to fix atmospheric nitrogen and promote plant growth (Riggs et al. 2001). Minorsky (2008) demonstrated vigorous colonization of a PGP strain *Pseudomonas fluorescens* B 16 in the roots of tomato resulting in higher yield. In chickpea, fluorescent pseudomonads were reported to promote plant growth, nodulation, and nitrogen fixation (Parmer and Dadarwal 1999). Though a number of literatures are available for PGP of various functional groups of bacteria, the demonstration of PGP bacteria under on-station and on-farm field conditions are scarce. Therefore, the present investigation was aimed to characterize and evaluate a root nodule bacterium isolated from chickpea nodule for its ability to promote plant growth in chickpea under greenhouse, on-station and on-farm field conditions.

## Materials and methods

A bacterium designated as IC-76, acquired from microbial gene bank at ICRISAT, was used extensively in this study. IC-76 was originally isolated from the nodules of chickpea (*Cicer arietinum* L.) by ICRISAT from the alluvial soils of Haryana, India. The actinomycetes CAI-24 (*Streptomyces tsusimaensis*; NCBI Accession Number: JN400112), CAI-121 (*Streptomyces caviscabies*; NCBI Accession Number: JN400113), CAI-127 (*Streptomyces setonii*; NCBI Accession Number: JN400114), KAI-32 (*Streptomyces africanus*; NCBI Accession Number: JN400115) and KAI-90 (*Streptomyces* sp.; NCBI Accession Number: JN400116), reported earlier by us as potential for biocontrol traits against *Fusarium* wilt in chickpea (Gopalakrishnan et al. 2011c), were used in this investigation.

The bacterium was evaluated for production of indole acetic acid (IAA), β-1,3-glucanase, hydrocyanic acid (HCN), siderophore, cellulase, lipase, chitinase, protease, and P-solubilizing capacity. IC-76 was estimated for IAA production in yeast extract mannitol (YEM) broth supplemented with l-tryptophan (1 µg mL^−1^) as per the protocols of Patten and Glick (1996). β-1,3-glucanase was done as per the protocols of Singh et al. (1999) and Gopalakrishnan et al. (2014) in tryptic soy broth, supplemented with 1 % colloidal chitin (w/v). One unit of β-1,3-glucanase activity was defined as the amount of enzyme that liberated 1 µmol of glucose hour^−1^ at defined conditions.

HCN was qualitatively estimated by sulfocyanate method (Lorck 1948). In brief, IC-76 was grown in triplicates in YEM agar amended with glycine (4.4 g L^−1^). Sterilized Whatman filter paper no. 1 (one sheet of 8 cm diameter) was soaked in 1 % picric acid (in 10 % sodium carbonate) for a minute and stuck underneath the Petri plate lids. The Petri plates were sealed with parafilm and incubated at 28 °C for three days. Development of reddish brown color on the Whatman filter paper indicated HCN production. The experiment was conducted three times. Observations were noted on a 0−3 rating scale, based on the intensity of the reddish brown color, as follows: 0, no color; 1, light reddish brown; 2, medium reddish brown, and 3, dark reddish brown.

Siderophore production was assessed in King’s B agar as per the protocol of Schwyn and Neilands (1987). The standardized protocols of Hendricks et al. (1995) were used in cellulose congo red agar to evaluate the cellulase production. The lipase and protease production was estimated as per the protocols of Bhattacharya et al. (2009) in tween 80 agar and casein agar, respectively. Chitinase production was determined in minimal media with 5 % colloidal chitin as per the protocols of Hirano and Nagao (1988). All the experiments were conducted three times in triplicated treatments. Observations of the IC-76 to siderophore, cellulase, lipase, and protease were noted on a 0−4 rating scale as follows: 0, no halo zone; 1, halo zone of <1 mm; 2, halo zone of 1−3 mm; 3, halo zone of 4−6 mm; 4, halo zone of 7−9 mm, and above. Chitinase production was noted on a 0−5 rating scale as follows: 0, no halo zone; 1, halo zone of 1−5 mm; 2, halo zone of 6−10 mm; 3, halo zone of 11−15 mm; 4, halo zone of 16−20 mm, and 5, halo zone of 21 mm, and above.

IC-76 was screened for its phosphate solubilizing ability on Pikovskaya agar (Pikovskaya 1948). The bacterium was streaked on Pikovskaya agar and incubated at 28 °C for three days. The presence of halo zone around the bacterium indicated positive.

IC-76 was evaluated for their PGP potential in greenhouse on chickpea as mono-culture and as co-inoculation with five strains of *Streptomyces* sp., such as CAI-24, CAI-121, CAI-127, KAI-32, and KAI-90. A total of eight treatments (IC-76, IC-76 + CAI-24, IC-76 + CAI-121, IC-76 + CAI-127, IC-76 + KAI-32, IC-76 + KAI-90, IC-76 + the consortia of five *Streptomyces* sp. and un-inoculated control) were made with six replications. Pot mixture (1000 g) was prepared by mixing black soil, sand, and farmyard manure at 3:2:2 and placed in 20 cm diameter plastic pots. Chickpea seeds (Kabuli variety ICCV-2 which matures at 85–90 days) were surface sterilized with sodium hypochlorite (2.5 % for 5 min) and rinsed thoroughly with sterilized water. The sterilized seeds were transferred into IC-76 culture broth (10^8^ CFU mL^−1^; grown in YEM broth separately) and incubated for 50 min. At the end of incubation, where required, the seeds were further transferred into the respective *Streptomyces* strains (10^8^ CFU mL^−1^; grown in starch casein broth separately) for 50 min before being sown in the pots (three seeds/pot but thinned to one after one week). Booster doses of IC-76/*Streptomyces* strains (5 mL per pot, 10^8^ CFU mL^−1^) were applied at 15, 30, 45, and 60 days after sowing by soil drench method. Plants were irrigated once every three days with 30 mL sterilized distilled water. PGP parameters, including plant height, leaf area and weight, nodule number and weight, stem weight and root weight, length and volume, were determined at day 30 after sowing. At harvest, pod weight and number and seed weight and number were recorded. Soil samples were collected (0–15 cm soil profile) at harvesting and analyzed for soil chemical traits including total N, available P, and  % organic carbon as per the protocols of Novozamsky et al. (1983), Olsen and Sommers (1982) and Nelson and Sommers (1982), respectively.

IC-76 was evaluated for PGP potential under on-station field conditions. The experiment was conducted in 2013–14 cropping seasons (post-rainy) at ICRISAT, Patancheru, Andhra Pradesh, India with chickpea variety ICCV-2, which normally yields 1.1−1.2 t ha^−1^. Soils at the experimental site are classified as Vertisol (fine montmorillonitic isohyperthermic typic pallustert) having 26 % sand, 21 % silt, and 52 % clay with alkaline pH of 7.7−8.6 and organic carbon content of 0.4−0.6 %. The mineral content of the top 15 cm rhizosphere soil include 24.7 mg kg^−1^ soil of available nitrogen, 8.6 mg kg^−1^ soil of available phosphorus and 298 mg kg^−1^ soil of available potassium. The experiment was laid out in a randomized complete block design (RCBD) with three replicates and subplot sizes of 4 m × 3 ridges. IC-76 was grown on YEM broth at 28 °C for three days and further evaluated for their PGP traits. The control plots contained no IC-76 strain. Chickpea seed was treated with IC-76 (containing 10^8^ CFU mL^−1^) for 50 min and sown on 1st November 2013 at a row-to-row spacing of 60 cm and a plant-to-plant spacing of 10 cm. IC-76 (1,000 mL; 10^8^ CFU mL^−1^) was applied once in 15 days on the soil close to the plant until the flowering stage. Irrigation was done on 21 days and 49 days after sowing. Weeding was done as and when required. No serious insect pests or phytopathogen attack were observed during the cropping period. The crop was harvested manually on 6th Feb 2014. At 30 days after sowing, plant height, nodule number, and weight and shoot and root weight whereas at harvest stover yield (t ha^−1^), grain yield (th a^−1^), total dry matter (th a^−1^), pod weight (g plant^−1^), seed number (plant^−1^), and 1000 seed weight (g) were noted. Soil samples were collected from 0–15 cm soil profile at flowering and harvest stages and analyzed for soil chemistry [total nitrogen (ppm), available phosphorus (ppm) and  % organic carbon as per the protocols of Novozamsky et al. (1983); Olsen and Sommers (1982) and Nelson and Sommers (1982), respectively].

IC-76 was also evaluated under on-farm field conditions at Mennipadu and Ramapuram villages (220 and 214 km, respectively, from ICRISAT) of Mahbubnagar districts of Andhra Pradesh, India. The experiment was conducted at four farmer’s field (one acre each) in each village in 2013–14 post-rainy seasons with Kabuli chickpea variety ICCV-2 onVertisol under rain fed conditions. The crop was sowed on 15th October 2013 and harvested manually on 21st January 2014. All the agronomic practices were followed as per on-station field conditions. Plant growth parameters including stover + pod yield (t ha^−1^), grain yield (t ha^−1^), shoot and pod weight (g plant^−1^), number of pods (plant^−1^), and number of seeds (plant^−1^) were determined at harvest.

For molecular identification of IC-76, pure culture of IC-76 was grown in YEM broth until log phase (three days). Genomic DNA was isolated as per the protocols of Bazzicalupo and Fani (1995) whereas 16S rDNA gene was amplified using universal primer 1492R (5’-TAC GGY TAC CTT GTT ACG ACT T-3’) and 27F (5’- AGA GTT TGA TCM TGG CTC AG-3’) as per the conditions described by Pandey et al. (2005). The PCR product was sequenced at Macrogen Inc. Seoul, Korea. The sequences obtained from Macrogen Inc. were compared with those from the GenBank using the BLAST program (Alschul et al. 1990), aligned using the Clustal W software (Thompson et al. 1997) and phylogenetic trees inferred using the MEGA version 4 program (Tamura et al. 2007) by neighbor-joining method (Saitou and Nei 1987). The nucleotide sequences of IC-76 were submitted to GenBank and the NCBI GenBank accession number was obtained.

For statistical analysis, the data were subjected to Analysis of Variance (ANOVA; GenStat 10.1 version 2007, Lawes Agricultural Trust, Rothamsted Experimental Station) to evaluate the efficiency of PGP agent’s application in the greenhouse and field studies. Significance of differences between the treatment means was tested at *P* < 0.001, 0.01, and 0.05.

## Results and discussion

Plant growth-promoting microbes having the ability to fix biological nitrogen would have an advantage in counter-balancing the loss of nitrogen from soils. In the present investigation, a bacterium (IC-76) isolated from the nodules of chickpea was characterized for in vitro PGP properties and further evaluated in the greenhouse and on-station and on-farm field conditions for PGP activity in chickpea. PGP microbes enhance host plant growth by exploiting different mechanisms. In the present study, IC-76 produced IAA, siderophore, HCN, cellulase, protease, and β-1,3-glucanase while it did not produce lipase and chitinase and solubilize phosphorus (Table 1). Among the PGP attributes studied, IC-76 produced large volumes of IAA (327 µg mL^−1^) and siderophore (45 % units). IAA producing bacteria are known to stimulate seed germination, initiate lateral and adventitious root formation and increase root surface area and length thereby provides the host plant greater access to water and soil nutrients (Ahemad and Kibret 2014). Siderophores act as solubilizing agents for iron from minerals under conditions of iron limitation (Indiragandhi et al. 2008). In addition, siderophores form stable complexes with heavy metals, including U, Np, Al, Cu, Cd, In, Ga, Zn, and Pb and increases the soluble metal concentration (Rajkumar et al. 2010), thus, it help to alleviate the stresses imposed on plants by heavy metals in soils. HCN production by bacteria is reported to play a role in disease suppression, as in the case of tobacco where *Pseudomonas fluorescens* helped suppression of black root rot disease (Haas et al. 1991). Cellulase and protease-producing microorganisms play an important role in the organic matter decomposition, nutrient mineralization, and PGP (Lima et al. 1998). The cell wall of plant pathogenic fungi, for instance *Fusarium oxysporum*, is composed of layers of β-1,3-glucan and lysis of this by β-1,3-glucanase-producing bacteria leads to leakage of cell contents and collapse of the pathogenic fungi (Singh et al. 1999). It is concluded that the bacterial strain isolated from the chickpea rhizosphere contains multi-trait of PGP, and therefore, IC-76 can be exploited not only for PGP but also for biological control of plant pathogens and/or degradation of organic residues.

**Table 1:** In vitro PGP traits of IC-76

Trait	Concentration/rating	SE
Indole acetic acid (IAA; µgml^−1^)	327.0	7.350
Siderophore (% units)	44.7	0.220
Hydrocyanic acid (HCN)	2.0	0.000
Cellulase	2.0	0.030
Lipase	0.0	0.000
Chitinase	0.0	0.000
Protease	2.0	0.000
β-1,3-Glucanase (% units)	0.9	0.003
P-solubilization	0.0	0.000

*SE* standard error

When IC-76 evaluated for its PGP potential under green house, at 30 days after sowing, IC-76 significantly enhanced PGP traits on leaf area (up to 9 %), root weight (up to 9 %), length (up to 20 %), and volume (up to 10 %) and stem weight (up to 7 %) over un-inoculated control. Besides this, IC-76 either in combination with individual *Streptomyces* sp. or with consortia of *Streptomyces* sp. significantly enhanced all the PGP traits including plant height (up to 11 %), leaf area (up to 35 %), leaf weight (up to 133 %), nodule number (up to 103 %) and weight(up to 181 %), root weight (up to 35 %), length (up to 109 %), and volume (up to 49 %; Table 2). Similar trend was observed at harvest as well where IC-76 in combination with individual *Streptomyces* sp. or consortia of *Streptomyces* sp. significantly enhanced the PGP traits of chickpea than the application of IC-76 alone (Table 3). At harvest, IC-76 as individual and with consortia of *Streptomyces* sp. significantly enhanced total N (up to 22 %), available P (up to 59 %), and  % organic carbon (up to 8 %) over un-inoculated control. However, when IC-76 was combined with individual *Streptomyces* sp., only available phosphorus was enhanced by all five treatments whereas for total N and  % organic carbon, only three of the treatments (CAI-24 + IC-76, CAI-121 + IC-76, and KAI-32 + IC-76) could enhance (Table 3). Though the IC-76 enhanced chickpea plant growth significantly the enhancement was even greater when it was combined with the five *Streptomyces* sp. Valverde et al. (2006) observed increased chickpea growth performance and seed yield by 52 % when *P. jessenii* PS06 co-inoculated with *Mesorhizobium ciceri* C-2/2 under greenhouse and field conditions. Dey et al. (2004) recorded enhanced yield performance and nodule weight on peanut by a consortium of *P. fluorescens* strains PGPR1, PGPR2, and PGPR4. Root development of plants plays key role in nutrient management and enhanced plant growth. The five *Streptomyces* sp. used in this study were also reported earlier to have PGP traits in both sorghum as well as rice (Gopalakrishnan et al. 2013b). Hence, it is concluded that both IC-76 and the five *Streptomyces* sp. has the potential for PGP in chickpea.

**Table 2:** Effect of IC-76, as individual culture and co-inoculation with PGP actinomycetes, on growth performance of chickpea at 30 days after sowing under greenhouse conditions

Treatments	Plant height (cm)	Leaf area (cm^2^)	Leaf weight (g)	Nodule number	Nodule weight (mg)	Root weight (mg)	Stem weight (mg)	Root length (cm)	Root volume (cm^3^)
Control	24.7	65	0.30	7.7	3.1	87	108	552	1.96
IC-76	24.8	71	0.30	7.8	3.1	95	115	660	2.15
CAI-24 + IC-76	27.3	79	0.36	11.0	6.5	117	142	723	2.76
CAI-121 + IC-76	26.3	71	0.31	8.2	4.2	103	192	951	2.44
CAI-127 + IC-76	26.8	81	0.51	7.8	3.0	107	243	840	2.43
KAI-32 + IC-76	27.3	82	0.51	11.0	5.2	105	295	894	2.52
KAI-90 + IC-76	27.2	82	0.49	8.3	3.2	112	263	704	2.36
Consortia + IC-76	24.8	88	0.70	15.6	8.7	105	368	1156	2.91
Mean	26.2	77	0.44	9.7	4.6	104	206	810	2.44
SE±	0.60**	3.1**	0.028*	0.45***	0.41***	4.1**	12.7***	29.3***	0.118***
LSD (5 %)	1.83	9.5	0.085	1.36	1.24	12.3	38.7	89.0	0.358
CV %	4	7	11	8	15	7	9	6	9

*SE* standard error, *LSD* least significant difference, *CV* coefficient of variance

** Statistically significant at 0.01

*** Statistically significant at 0.001

**Table 3:** Effect of *P. geniculata* IC-76, as individual culture and co-inoculation with PGP actinomycetes, on yield performance of chickpea and soil nutrient traits at harvest stage under greenhouse conditions

Treatments	Pod weight (g)	Pod number	Seed weight (g)	Seed number	Total N (ppm)	Available P (ppm)	% Organic carbon
Control	5.94	23	3.5	23	2153	67.4	2.14
IC-76	7.09	26	5.4	29	2562	71.6	2.25
CAI-24 + IC-76	7.78	33	6.0	33	2620	73.6	2.42
CAI-121 + IC-76	9.06	35	6.2	35	2942	98.6	2.64
CAI-127 + IC-76	7.84	30	6.3	30	2062	74.2	1.76
KAI-32 + IC-76	7.46	30	5.6	30	3356	103.3	2.56
KAI-90 + IC-76	7.29	27	5.6	27	2053	93.3	1.73
Consortia + IC-76	8.73	36	6.7	36	2620	106.8	2.31
Mean	7.65	30	5.7	30	2546	86.1	2.22
SE±	0.197***	0.9***	0.22***	1.1***	133.2**	1.85***	0.060***
LSD (5 %)	0.659	3.2	0.73	3.6	445.5	6.19	0.199
CV %	4	5	5	5	7	3	4

*SE* standard error, *LSD* least significant difference, *CV* coefficient of variance

** Statistically significant at 0.01

*** Statistically significant at 0.001

Under on-station field conditions, at 30 days after sowing, IC-76 significantly enhanced plant height (10 %), nodule number (6 %) and weight (6 %), root weight (15 %) and shoot weight (21 %) while at harvest, stover yield (45 %), grain yield (14 %) and total dry matter (29 %) over the un-inoculated control (Tables 4, 5). The total N, available P, and  % organic carbon were significantly higher in the top 15 cm of rhizosphere soils of IC-76 treated plants by 5, 4, and 2 %, respectively, at flowering stage and by 3, 16, and 6 %, respectively, at harvesting stage over the un-inoculated control (Table 6). Under on-farm field conditions, at Mennipadu and Ramapuram villages, IC-76 significantly enhanced stover yield (up to 43 and 33 %, respectively), grain yield (up to 59 and 31 %, respectively), shoot weight (up to 91 and 12 %, respectively), pod weight plant^−1^ (up to 71 and 50 %, respectively), pod numbers plant^−1^ (up to 59 and 24 %, respectively), and seed number plant^−1^ (up to 69 and 39 %, respectively) over un-inoculated control in all the four farmer’s fields (Table 7).

**Table 4:** Effect of IC-76 for their PGP potential in chickpea under on-station field conditions—at 30 days after sowing

Treatments	Plant height (cm)	Nodule number (plant^−1^)	Nodule weight (mg plant^−1^)	Root weight (mg plant^−1^)	Shoot weight (g plant^−1^)
IC-76	32	52	235	193	2.08
Control	29	49	222	168	1.72
Mean	31	51	229	181	1.90
SE±	0.4*	0.4*	0.8**	3.8*	0.025**
LSD (5 %)	2.5	2.5	5.0	23.1	0.149
CV %	2	1	1	4	2

*SE* standard error, *LSD* least significant difference, *CV* coefficient of variance

* Statistically significant at 0.05

** Statistically significant at 0.01

**Table 5:** Effect of IC-76 for their PGP potential in chickpea under on-station field conditions—at harvest

Treatments	Stover yield (t ha^−1^)	Grain yield (t ha^−1^)	TDM (t ha^−1^)	Pod weight (g plant^−1^)	Seed number (plant^−1^)	1000 seed weight (g)
IC-76	2.43	2.01	4.44	21.92	82	198
Control	1.68	1.77	3.44	21.65	80	197
Mean	2.05	1.89	3.94	21.78	81	197
SE±	0.073*	0.011**	0.073**	0.028*	0.4*	0.3*
LSD (5 %)	0.445	0.066	0.442	0.172	2.2	1.4
CV %	6	1	3	1	1	1

*TDM* total dry matter, *SE* standard error, *LSD* least significant difference, *CV* coefficient of variance

* Statistically significant at 0.05

** Statistically significant at 0.01

**Table 6:** Effect of IC-76 on soil nutrient properties and biological activities at flowering and harvest stages of chickpea under on-station field conditions

At flowering stage				At harvest stage		
Treatments	Total N (ppm)	Available P (ppm)	Organic carbon (%)	Total N (ppm)	Available P (ppm)	Organic carbon (%)
IC-76	707	9.6	0.55	759	8.0	0.58
Control	675	9.2	0.54	735	6.9	0.55
Mean	691	9.4	0.54	747	7.4	0.57
SE±	1.4*	0.02*	0.001*	1.4*	0.05*	0.002*
LSD (5 %)	25.4	0.32	0.013	25.4	0.95	0.032
CV %	1	1	1	1	1	1

*SE* standard error, *LSD* least significant difference, *CV* coefficient of variance

* Statistically significant at 0.05

**Table 7:** Effect of IC-76 on yield performance of chickpea under on-farm field conditions at Mennipadu and Ramapuram villages

	Stover + pod yield (t ha^−1^)		Grain yield (t ha^−1^)		Shoot weight (g plant^−1^)		Pod weight (g plant^−1^)		Number of pods (plant^−1^)		Number of seeds (plant^−1^)	
Farmer	Cont.	Inoc.	Cont.	Inoc.	Cont.	Inoc.	Cont.	Inoc.	Cont.	Inoc.	Cont.	Inoc.
Mennipadu												
1	2.54	3.57	1.40	2.23	2.15	3.05	6.8	11.6	27	43	26	44
2	2.84	4.05	1.92	2.13	2.19	4.18	7.0	9.5	27	38	27	40
3	2.96	3.81	1.78	2.41	3.33	3.56	8.2	11.4	33	40	34	41
4	3.57	4.31	1.94	2.54	3.41	3.76	9.2	9.5	35	37	36	37
Mean	2.98	3.93	1.76	2.33	2.77	3.64	7.8	10.5	30	40	31	41
SE±	0.035***		0.035***		0.126***		0.10***		0.4***		0.3***	
CV %	4		6		14		4		4		3	
Ramapuram												
1	4.70	6.03	2.86	3.25	8.30	8.68	17.6	21.2	50	60	51	60
2	4.98	6.62	3.03	3.96	4.29	4.80	16.5	18.9	48	57	47	56
3	5.11	6.62	3.27	3.74	5.03	5.30	19.9	20.2	54	56	55	57
4	5.50	6.37	2.65	3.48	6.38	7.06	13.1	19.6	38	47	39	54
Mean	5.07	6.41	2.95	3.61	6.00	6.46	16.8	19.9	48	55	48	57
SE±	0.047***		0.036***		0.031***		0.36***		0.6***		0.5***	
CV %	2		4		2		7		4		3	

*Cont*. control, *Inoc*. inoculated with IC 76 strain, *SE* standard error, *CV* coefficient of variance

*** Statistically significant at 0.001

In the present investigation, under on-station chickpea field conditions, IC-76 significantly enhanced plant height, nodule number and weight, root and shoot weight, stover yield (45 %), grain yield (14 %), and total dry matter (29 %) over the un-inoculated control (Table 5). Similar results were found when IC-76 was evaluated on chickpea at on-farm conditions at Mennipadu and Ramapuram villages of Andhra Pradesh, India where stover yield (up to 43 and 33 %, respectively) and grain yield (up to 59 and 31 %, respectively) were enhanced over the un-inoculated control (Table 7). The mechanism by which the IC-76 enhanced the PGP on chickpea could be their PGP traits including IAA and siderophore production (direct simulation of PGP) and HCN, cellulase, protease, and β-1,3-glucanase (indirect stimulation of PGP; Table 1). The effect of bacteria on PGP including root development has been widely reported (Birkhofer et al. 2008; El-Tarabily 2008; Uphoff et al. 2009; Gopalakrishnan et al. 2012a, 2013a, 2014). In the present investigation, although, chickpea roots were not inspected for colonization, the repeated data on root morphology (including root weight, length, and volume) and soil chemical activities (including  % organic carbon, total N, and available P) in greenhouse and on-station field conditions strongly suggest that the IC-76 had multiplied and colonized the roots of chickpea plants. Mercado-Blanco and Bakker (2007) reported series of research work on *P. fluorescens* WCS365 ability to colonize plant roots. Therefore, it can be concluded that the IC-76 was able to survive in the chickpea rhizosphere, colonize on the chickpea roots, and promote PGP and enhance soil health.

**Fig. 1:**
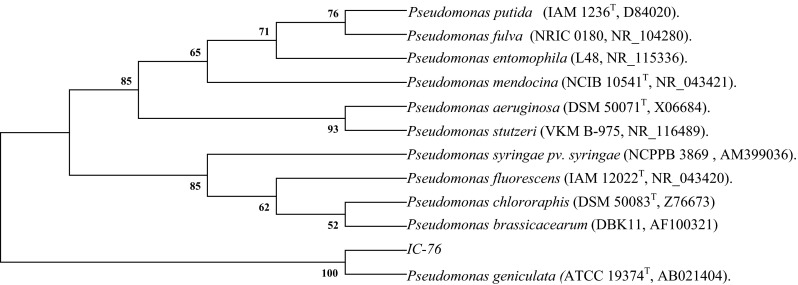
Phylogenetic relationship between IC-76 and representative species based on full length 16S rDNA sequences constructed using the neighbor-joining method

To identify the IC-76, its 16S rDNA was sequenced and analyzed. A neighbor-joining dendrogram was generated using the sequence from IC-76 (857 bp) and representative sequences from the databases. Phylogenetic analysis of 16S rDNA sequence of IC-76 showed maximum sequence similarity (100 %) with *Pseudomonas geniculata* (Fig. 1). When the nucleotide sequences were submitted, GenBank assigned NCBI accession number KM 581022 for IC-76. Several species of *Pseudomonas* such as *P. aeruginosa* (Ganesan 2008), *P. fluorescens* (Shaharoona et al. 2006), *P. putida* (Tripathi et al. 2005), *P. jessenii* (Rajkumar and Freitas 2008), and *P. chlororaphis* (Liu et al. 2007) also have been documented for one or multiple PGP traits. Enhanced plant growth performance and soil nutrient parameters has been observed in pot studies on various *Pseudomonas* sp., as individual culture on (i) mung bean by *Pseudomonas* sp. (Gupta et al. 2002), *P. putida* KNP9 (Tripathi et al. 2005); (ii) soybean by *P. fluorescens* (Gupta et al. 2005) and (iii) black gram by *P. aeruginosa* (Ganesan 2008). *Mesorhizobium* sp. and *Bacillus* sp. and even *Pseudomonas* sp. have been demonstrated for their PGP in chickpea under greenhouse conditions (Parmer and Dadarwal 1999; Tank and Saraf 2009; Ahemad and Khan 2010; Wani and Khan 2010), however, as of the author’s knowledge; there are no reports of *P. geniculata* demonstrated for PGP potentials in chickpea or any other crop under field conditions. Hence, this is the first report acknowledging PGP potential of *P. geniculata* under laboratory, green house, on-station and on-farm field conditions on chickpea. The PGP traits of the *P. geniculata* IC-76 and its effect on chickpea plant growth and yield suggest that this strain has a potential of being developed as bio-inoculant and hence is more likely to be a promising strain for application in agriculture under semi-arid conditions.
